# Effects of Dust Bath Design on Hen Behavior in New Aviary Systems in China

**DOI:** 10.3390/ani15202946

**Published:** 2025-10-10

**Authors:** Zhihao Zhang, Qian Zhang, Jianying Xu, Baoming Li, Weichao Zheng, Yang Wang

**Affiliations:** 1 Department of Agricultural Structure and Environmental Engineering, College of Water Resources and Civil Engineering, China Agricultural University, Beijing 100083, China; zhangzhihao0027@163.com (Z.Z.);; 2 Key Laboratory of Agricultural Engineering in Structure and Environment, Ministry of Agriculture and Rural Affairs, Beijing 100083, China; 3 Beijing Engineering Research Center on Animal Healthy Environment, Beijing 100083, China

**Keywords:** poultry, welfare, aviary, dustbathing, substrate depth

## Abstract

**Simple Summary:**

An important behavior of laying hens is dustbathing, which generally occurs in areas covered with litter. Extensive litter areas commonly used in aviaries can lead to reduced air quality and increased incidence of diseases, making them unsuitable for deployment in new large cage aviary unit systems. Using dust baths to provide dustbathing materials is a better option because they offer advantages in terms of continuous availability, but their design lacks unified standards. This study examined how different areas, shapes of dust baths, and depths of dustbathing materials affect the dustbathing behavior of hens. After increasing the dust bath areas, the daily proportion of dustbathing hens did not increase. Both the dust bath shape and the dustbathing material depth could affect the dustbathing expression. The hens exhibited better dustbathing behavior in circular dust baths and with 5 cm deep dustbathing materials. These findings can provide a basis for the design of dust baths in new aviary systems.

**Abstract:**

Alternative housing systems for laying hens, such as the aviary, promote the expression of dustbathing behavior by providing substrate materials to improve their welfare. However, extensive litter areas in aviaries can lead to reduced air quality and increased incidence of diseases, making them unsuitable for deployment in new large cage aviary unit (LCAU) systems in China. Dust baths have advantages in terms of continuous availability, but their design lacks unified standards. This study explored the effects of different areas, shapes (circular and square), and substrate depths (1 cm, 5 cm, 9 cm) of dust baths on dustbathing behavior in LCAU systems by recording digital video. Each LCAU system was initially populated with 305 Jingfen No. 2 laying hens at 50 days of age. The dust baths were initially placed on the bottommost tier at 66 days of age. The results showed that after approximately 3 weeks of adaptation to dustbathing, the average daily proportion of dustbathing hens within the flock stabilized at approximately 10%. A 50 cm diameter circular dust bath could accommodate their dustbathing requirements. Increasing the number of circular dust baths to 2 did not significantly affect the daily proportion of dustbathing hens. Both the circular dust bath and a 5 cm depth substrate resulted in better expression of the hens’ side rubbing behavior and the lower frequency of tossing behavior.

## 1. Introduction

With the drawbacks of conventional cage systems for laying hens becoming increasingly prominent, growing attention is being paid to animal health and welfare [1,2,3]. During the transition of the Chinese laying hen industry from conventional cage systems to welfare-friendly production systems, China Agricultural University had developed the large cage aviary unit (LCAU) system for laying hens that contains perches and nest boxes [4]. Commonly used aviaries abroad contain open floor litter areas, which can promote hens to perform behaviors such as dust bathing, pecking, and scratching [5]. However, the management of litter areas presents significant challenges, as feces-contaminated bedding is often not replaced promptly. This makes litter the dominant source of PM (particulate matter) and other air pollutants, and increases the incidence of diseases in chickens and the occurrence of mite infestations [6,7,8,9]. Therefore, the multi-tier LCAU system does not use litter, resulting in a significantly lower indoor PM concentration compared to the values reported for other aviary systems [4,10].

However, dustbathing is a functionally important behavior for laying hens, as it can remove fatty agents and ectoparasites from the feathers and realign feather structure [11,12]. Hens are highly motivated to dustbathe and will sham dustbathing on the wire mesh when they have no access to suitable dustbathing material [13]. To avoid compromising the welfare of hens, it is necessary to provide dustbathing materials. Considering animal welfare, indoor air quality, and management feasibility, providing an area covered with dustbathing substrate instead of an extensive litter floor in the LCAU is a more feasible strategy to promote the dustbathing behavior of laying hens.

There are two main methods for providing dustbathing substrate. One is placing a mat over the wire floor, which consists of sections of synthetic turf or other artificial materials [14,15]. However, the substrate placed on the mat is often insufficient, as pecking, scratching, and raking motions during dust bathing accelerate substrate dispersion through the wire mesh, necessitating frequent substrate replenishment. Auger mechanisms are typically equipped to deliver additional dustbathing substrate onto the mat. The substrate distributed type must be compatible with the distribution system and satisfy the hens. However, materials commonly used for laying hens, such as sand, peat, wood shavings, sawdust, and straw, are incompatible with auger mechanisms, because they can cause clogging or wear of the mechanisms [16]. Currently, a common practice in poultry farms is to use food particles as substrate materials [17], as they can be easily distributed without requiring an additional system. However, food particles may not be a suitable substrate for dustbathing in laying hens due to their high lipid content [18].

The other method involves using dust baths to contain the substrate [19]. The dust baths can accommodate greater substrate depths and reduce the substrate dispersion rate, but may induce mislaid eggs outside the nest. Due to their advantage over mats in terms of continuous availability and their ability to improve air quality compared to litter areas [7,10,20], dust baths are more suitable for application in LCAU systems. Unified standards for the shape and substrate depth of dust baths are lacking. In dustbathing studies to date, widely varying substrate depths have been used, ranging between 1 cm [21] and 20 cm [22]. Baumann [23] recommends a substrate depth of 10 cm. Moesta et al. [22] indicated that substrate depth had a limited influence on the expression of dustbathing behavior in laying hens. However, it interacted with substrate quality, and hens performed more vertical wing shakes on 2 cm deep substrates, possibly because it was more difficult to toss the substrate into their feathers.

In addition to design, the size of the dust bath area also affects the expression of dustbathing behavior in hens. Louton et al. [24] observed a positive correlation between the size of the dust bath area and dustbathing activity. In contrast, small and subdivided dust bath areas reduced the dustbathing frequency and increased the incidence of sham dustbathing on wire mesh floors. Van Liere [25] found that the duration of dustbathing in hens was longer on wider mats compared to several smaller ones, and the dustbathing bouts were incomplete if the dust bath area provided was too small. Beyond the requirement of individual hens for dustbathing space, the size of the dustbathing area also depends on the proportion of dustbathing hens in the flock. There are individual differences in hens’ acceptance of dustbathing facilities. Roll et al. [26] observed that in enriched cages, the maximum proportion of hens present at the dustbathing area was 21.4%, and the maximum proportion of hens performing dustbathing was 21.0%. Wall et al. [27] reported that 30% of hens in enriched cages never entered the dustbathing facilities.

The objective of this study was to explore the effects of the area, shape, and substrate depth of dust baths on the dustbathing behavior of laying hens, and thereby provide a basis for determining the design of dust baths matching the LCAU system and provide references for the optimization of dust bath area in alternative systems.

## 2. Materials and Methods

### 2.1. Hens and Housing System

**Figure 1:**
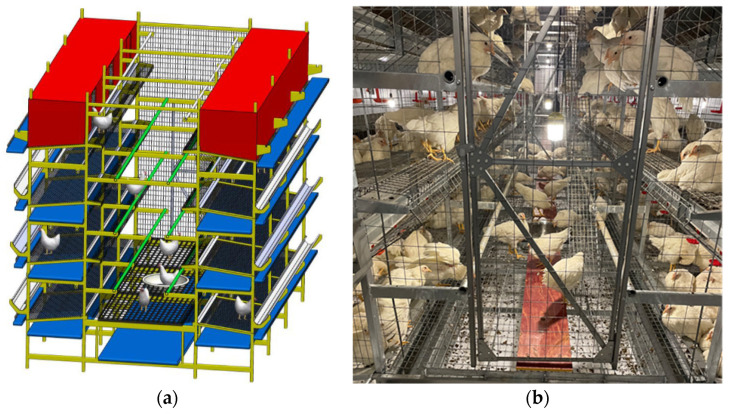
Schematic of the LCAU system. 3D schematic (**a**) and actual image (**b**).

Jingfen No. 2 laying hens were sourced from a single rearing farm and placed into LCAU systems at 50 days of age. Each LCAU system was initially populated with 305 hens. The LCAU system was symmetrically distributed, consisting of two rows of cages and a 1.3 m wide aisle in the middle (Figure 1). Each row contained 3 four-tier stacked cages, and the dimensions of a cage were 1.2 m × 0.7 m × 0.7 m. Each tier was equipped with nipple drinkers, manure belts, and rotating mesh doors for vaccination management. During the experiment, the rotating mesh doors were kept open, allowing all hens to move freely within the LCAU system. The bottom three tiers were equipped with feed troughs, and the nests were equipped on the top tier. Multi-tiered perches were installed in the aisle to enable vertical movement of the hens. Automatic feeding was performed 3 times daily at 6:30, 10:30, and 18:30. Water was provided ad libitum. The lighting period lasted from 4:30 to 20:30 daily (16L:8D).

### 2.2. Experimental Design

Experiments were conducted in three LCAU systems equipped with dust baths to investigate the effects of dust bath area and shape, and dustbathing substrate depth on the dustbathing behavior of laying hens, respectively. In each LCAU system, only one target factor was modified while maintaining consistent non-target factors and husbandry conditions. The dust baths were initially placed on the bottommost tier at 66 days of age, and sand was selected for the substrate [28] and replenished daily to maintain the target depth. The hen behaviors were recorded with digital video cameras (CS-C2C-1B2WFR, Hangzhou Fluorite Software Co., Ltd., Hangzhou, China) installed above the dust bath. All data were collected by the same trained observer. For all trials, the experimental unit was the individual hen, and the sample size was more than 30 per test group [29].

#### 2.2.1. Dust Bath Area

The designed capacity of hens per dust bathing bout was calculated as follows:

(1) $$A=abtNA_{0}$$

where A is the size of the dust bath area, m^2^; a is the average daily fraction of dustbathing hens; b is the average hourly fraction of dustbathing hens during the concentrated dust bathing periods, h^−1^; t is the duration of a dust bathing bout, h; N is the hen number, hen; $A_{0}$ is the dust bath area requirement for a hen.

**Figure 2:**
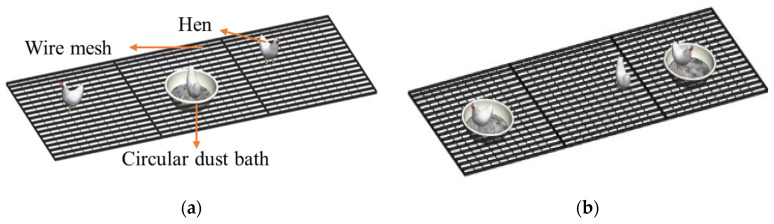
Layout of (**a**) 1 circular dust bath and (**b**) 2 circular dust baths on the bottommost tier of the LCAU system.

According to previous studies, a was 9.7% and b was 14.8% [20]. When the dust bath area is sufficient to allow unrestricted dust bathing for laying hens, the duration of a complete dust bathing bout is approximately 30 min. Therefore, the dust bath area in this study must be designed to accommodate at least 2 dustbathing hens. Based on the results found by Li [30], which indicate that $A_{0}$ for a Jingfen laying hen is 930 cm^2^, the size of the dust bath area should be no less than 1960 cm^2^. A circular dust bath with an inner diameter of 50 cm and a height of 17 cm was placed for 4 weeks, after which an additional identical dust bath was added for 2 weeks (Figure 2). The substrate depth was 5 cm.

Previous studies have observed that the hens perform dustbathing every second day [31]. Therefore, the average daily fraction of dustbathing hens was calculated in groups of 3 days. Record the start time and duration of dust bathing for each hen. A bout was considered to start when a hen squatted down and performed a vertical wing shake, and ended with the first body shake. If no such shake occurred, it ended at the start of a 5 min period without dustbathing behavior. The number of hens per simultaneous dustbathing occurrence was recorded. Using weeks as the statistical unit, the frequency of each hen count category was tallied, and its respective proportions were calculated.

#### 2.2.2. Dust Bath Shape

**Figure 3:**
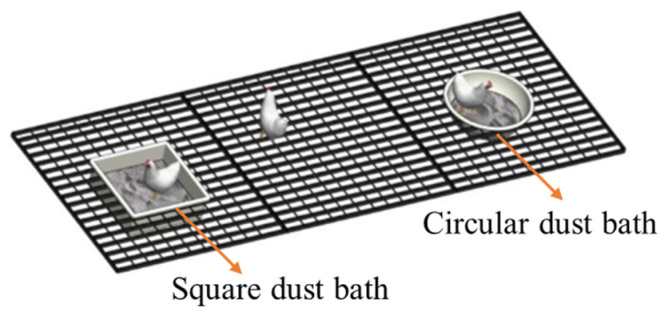
Layout of a square dust bath and a circular dust bath.

A square dust bath with dimensions of 50 cm long × 50 cm wide × 17 cm high and a circular dust bath were placed simultaneously in the LCAU system (Figure 3), and both were filled with 5 cm deep sand. After a habituation period of 3 weeks, video observation was conducted during 48 consecutive hours.

Dustbathing behaviour in fowl is functionally organized in sequences of tossing and rubbing behavior [32]. The tossing behavior, with elements such as vertical wing shaking or scratching with one leg, raises substrate onto the plumage and is performed with fluffed feathers, which facilitates the penetration of substrate. Rubbing includes side rubbing and is performed with the feathers flattened and the wings kept firmly to the body. Side rubbing was defined as the hen lying on one side and stretching its legs to rub its body against the substrate [22].

For each shape, complete dustbathing bouts in which laying hens dustbathed alongside the dust bath walls and performed side rubbing with legs close to the walls at initiation were selected to analyse the frequency of tossing behavior and the relevant data on side rubbing behavior, including the duration of leg stretch actions, the continuity and number of side rubbing bouts. Due to the short duration of a leg stretch action, the average duration was calculated by recording the total time of 5 consecutive leg stretch actions to reduce recording errors. Continuous side rubbing that was not interrupted by reinitiated tossing behavior was classified as a side rubbing bout. During each side rubbing bout, if the hen’s leg moved away from the dust bath walls, the bout was deemed discontinuous and scored 0; if the leg remained close to the dust bath walls, the bout was considered continuous and scored 1.

#### 2.2.3. Substrate Depth

A circular dust bath was placed in the LCAU system, and after a habituation period of 3 weeks, substrates with depths of 1 cm, 5 cm, and 9 cm were each provided for two days. During the complete dust bathing bouts at each substrate depth, the duration of leg stretch actions, the tossing behavior frequency, and the duration of dustbathing bouts were recorded.

### 2.3. Statistical Analysis

The data were organized using Microsoft Excel 2021 (Microsoft Corporation, Redmond, WA, USA) and statistically analysed with SPSS (version 26.0; IBM Crop., Armonk, NY, USA). All data were examined for normality (Kolmogorov–Smirnov test) and homogeneity of variance. Non-normal data were transformed with logarithm, arcsine, or square root to meet normality, followed by one-way ANOVA. *p* ≤ 0.05 presented as the difference is significant.

## 3. Results

### 3.1. Effects of Dust Bath Area on Dustbathing Behavior

**Figure 4:**
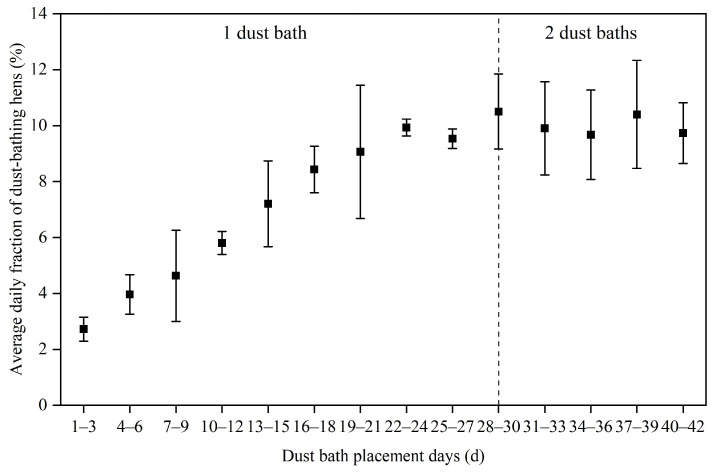
Average daily fraction of dustbathing hens with dust bath placement days.

The change trend in the average daily fraction of dustbathing hens in the LCAU system is shown in Figure 4. During the first 3 days after the dust bath was placed, the average daily fraction of dustbathing hens was 2.7%. Subsequently, this fraction increased at an average rate of 1.1% every 3 days, reaching 9.1% by days 19–21. After day 22, the daily fraction of dustbathing hens stabilized, fluctuating within the range of 9.5–10.5% with an average fraction of 10.0%. The highest fraction occurred between days 28–30, which was the early stage after the number of dust baths was increased to 2, with an average of 32 hens entering the dust baths to perform dust bathing behavior per day. However, the average daily fraction of dustbathing hens subsequently declined through days 31–36.

The proportions of hens dustbathing simultaneously in the dust baths are shown in Table 1. With 1 dust bath, a maximum of 5 hens were observed dust bathing simultaneously, and the proportion for instances of 2 hens was significantly the highest (*p* < 0.01). The proportion for 3 hens was markedly higher than for 4 or 5 hens (*p* < 0.01), while proportions for 4 and 5 hens showed no statistical difference (*p* > 0.05).

When two dust baths were provided, the maximum number of hens observed dustbathing simultaneously increased to 8, the proportion for 2 hens dustbathing simultaneously decreased by 18.49% and the proportion for 3 hens dustbathing simultaneously increased by 7.86% compared to when one dust bath was provided. However, increasing the number of dust baths did not change the proportional distribution pattern, which is that this proportion decreases as the number of co-dustbathing hens increases. The occurrence of 2 hens dustbathing simultaneously was significantly the highest (*p* < 0.01), followed by that of 3 hens dustbathing simultaneously (*p* < 0.01), while there was no significant statistical difference among the proportions of 4 to 8 hens dustbathing simultaneously (*p* > 0.05).

### 3.2. Effects of Dust Bath Shape on Dustbathing Behavior

The indicators of side rubbing and tossing behavior of laying hens in circular and square dust baths are shown in Table 2. The continuity score of side rubbing bouts in circular dust baths was 1.0 ± 0.0, meaning no leg movement away from the dust bath walls was observed in bouts initiated with the leg closer to the bath wall. Compared with the circular dust bath, in the square dust bath, the duration of a leg stretch action in laying hens increased by 50%, the number of side rubbing bouts decreased by 31.82%, and the tossing behavior frequency increased by 29.41%.

### 3.3. Effects of Substrate Depth on Dust Bathing Behavior

**Figure 5:**
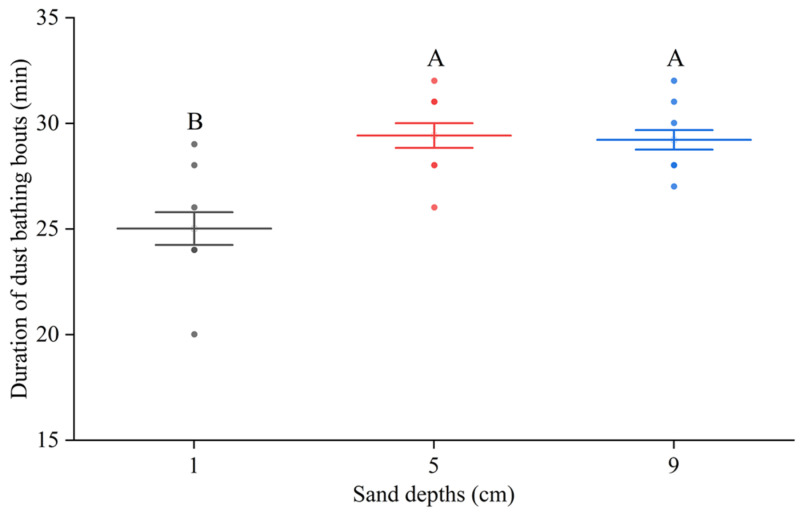
Duration of dust bathing bouts with various substrate depths. Different uppercase letters indicate significant differences (*p* < 0.01).

Table 3 presents the differences in the duration of a leg stretch action and the frequency of tossing behavior in laying hens under different sand depths. No significant difference emerged in leg stretch duration between 1 cm and 5 cm depths. When the sand depth increased to 9 cm, duration increased significantly to 1.0 ± 0.0 s. Tossing frequency was higher at 1 cm and 9 cm depths, while significantly decreasing at 5 cm depth. As shown in Figure 5, Dustbathing duration averaged 29.4 ± 0.6 min at 5 cm depth and 29.2 ± 0.5 min at 9 cm depth, with no significant difference. At 1 cm depth, dustbathing duration was significantly reduced to 25.0 ± 0.8 min (*p* < 0.01).

## 4. Discussion

Previous studies have mainly focused on the number and total fraction of hens present in the dust bath area [5,29], with little attention paid to the fraction of hens using the dust baths daily. However, in the LCAU system, laying hens can access the dust baths at any time and take turns to bathe. Therefore, the daily proportion of dustbathing hens is more suitable for accurately evaluating whether the area of the provided dust baths in the LCAU system can fulfill dustbathing behavioral needs. The laying hens in LCAU systems required 3 weeks to get used to the dust baths after they were placed. During this period, the daily proportion of dustbathing hens gradually increased. Dustbathing is socially facilitated and synchronous, resulting in the probability of dustbathing hens increasing when they observe other hens performing this behavior [33,34].

Based on the 2-day interval between dustbathing behaviors of hens and an average daily dustbathing proportion of 10%, it can be inferred that approximately 20% of the hens in this flock of 305 hens in the LCAU system regularly use the dust baths. This proportion is lower than the values reported by Louton et al. [24] and Roll et al. [26]. It is generally believed that in large cages with less adequate allowance of facilities per hen, high-ranking hens may have priority in using dust baths, while low-ranking hens may avoid the dust baths rather than compete for them.

However, in this study, although increasing the size of the dust bath area allowed more laying hens to dust bathe simultaneously, it did not lead to an increase in the fraction of dustbathing hens. This could be attributed to the impact of the dust baths’ location on the hens’ dustbathing motivation. The initiation of dustbathing behavior is also positively influenced by the sight of a dusty substrate [35]. Dust baths are placed on the first tier to reduce the height of dust dispersion and thereby minimize particulate generation, as well as to facilitate management. However, Yin et al. [36] reported that in the LCAU system, younger laying hens are mainly distributed in the upper tiers, where they are less likely to be exposed to the visual stimuli emanating from the substrate, consequently reducing the triggering of dustbathing behavior. Additionally, hens in the upper tiers need to jump downward to access the dust baths, so the vertical distance to the dust baths may affect the strength of their motivation to dustbathe, although this has not yet been studied. To balance the requirements of animal welfare, environmental control, and management efficiency, further research is necessary to determine the relationship between the proportion of hens from different tiers accessing the dust baths and the placement location of these facilities.

Both the dust bath shape and substrate depth affect the expression of hens’ tossing and side rubbing behaviors. Observations revealed arcuate claw trajectories of laying hens during leg stretch actions. Therefore, hens in a square dust bath were restricted by the bath wall during side rubbing with the wall-side leg and needed to move their legs away from the walls, resulting in lower continuity scores. The lower number of side rubbing bouts and slower leg stretch actions reflect a weaker motivation for side rubbing in hens in the square dust bath. The primary function of tossing behavior is to raise the substrate and distribute it among the feathers. Van Liere [25] reported that increased tossing behavior is positively correlated with difficulty in substrate movement and penetration within feathers. The higher tossing frequency in the square dust bath was attributed to the restriction of the bath walls on hens’ leg movements, compelling hens to perform more tossing actions to ensure sufficient substrate penetrates into the feathers. The curved sidewalls of the circular dust bath better accommodate the leg movement requirements of dustbathing hens.

When the substrate depth was 9 cm, laying hens formed deeper sand pits when lying down, resulting in their leg movements needing to push aside more sand, which caused greater movement resistance. Consequently, the increased difficulty of leg movements prolonged the duration of a leg stretch action and decreased the amount of substrate tossed per tossing action, leading to higher frequencies of tossing behavior. On shallow 1 cm substrates, where the material depth is less than the length of hens’ toes, thus making effective substrate tossing challenging, the hens also respond by increasing tossing frequency. These results are consistent with the report by Moesta et al. [22] that hens respond to the suboptimal depth of the substrate by increasing their efforts to enhance the contact between the feathers and the substrate. Longer duration of dustbathing is associated with a favorable perception of the fine particles in the substrate [12,37]. The shorter dust bathing duration of laying hens on the shallow substrate indicates a poorer perception of the substrate. Therefore, 5 cm depth sand allows laying hens to better perform dust bathing behavior compared to 1 cm and 9 cm depth sand.

## 5. Conclusions

In the LCAU system housing 305 hens, when the number of dustbathing hens stabilized, the average daily fraction of dustbathing hens in the flock was 10%, and their dustbathing requirements could be accommodated by a 50 cm diameter circular dust bath. Increasing the number of circular dust baths to 2 did not significantly affect the daily fraction of dustbathing hens. Circular dust bath with 5 cm deep sand enabled laying hens to perform dust bathing behavior more effectively compared to square dust bath and sand depths of 1 cm or 9 cm.

## Figures and Tables

**Table 1:** Proportions of hens dustbathing simultaneously under different numbers of dust baths.

Number of Hens Dustbathing Simultaneously	Proportion (%)	
	1 Dust Bath	2 Dust Baths
2	74.17 ± 1.84 ^A^	55.68 ± 9.83 ^A^
3	18.72 ± 0.75 ^B^	26.58 ± 5.57 ^B^
4	4.5 ± 1.5 ^C^	6.9 ± 3.67 ^C^
5	2.61 ± 1.56 ^C^	3.33 ± 2.49 ^C^
6	-	2.8 ± 1.5 ^C^
7	-	2.49 ± 1.42 ^C^
8	-	2.26 ± 1.51 ^C^
Maximum number	5	8

^A–C^ Different superscript uppercase letters within the same column indicate significant differences (*p* < 0.01).

**Table 2:** Differences in side rubbing and tossing behavior indicators of laying hens between circular and square dust baths.

Indicators	Circular Dust Bath	Square Dust Bath
Duration of a leg stretch action (s)	0.6 ± 0.1 ^A^	0.9 ± 0.0 ^B^
Continuity score of side rubbing bouts	1.0 ± 0.0 ^A^	0.4 ± 0.2 ^B^
Number of side rubbing bouts	2.2 ± 0.2 ^a^	1.5 ± 0.2 ^b^
Tossing behavior frequency (times/min)	3.4 ± 0.3 ^b^	4.4 ± 0.3 ^a^

^A,B,a,b^ Different superscript lowercase letters within the same row indicate significant differences (*p* < 0.05), and different uppercase letters indicate extremely significant differences (*p* < 0.01). The continuity score was operationally defined as: 0 indicating leg movement away from the dust bath walls, and 1 indicating no leg movement away during a side rubbing bout.

**Table 3:** Differences in side rubbing and tossing behavior indicators of laying hens with various substrate depths.

Substrate Depth (cm)	Duration of a Leg Stretch Action (s)	Tossing Behavior Frequency (times/min)
1	0.5 ± 0.0 ^B^	4.4 ± 0.2 ^A^
5	0.6 ± 0.0 ^B^	3.3 ± 0.2 ^B^
9	1.0 ± 0.0 ^A^	4.5 ± 0.2 ^A^

^A,B^ Different superscript uppercase letters within the same row indicate significant differences (*p* < 0.01).

## Data Availability

Data will be made available on reasonable request from the corresponding author.

## References

[1] Campbell D.L.M., de Haas E.N., Lee C. (2019). A review of environmental enrichment for laying hens during rearing in relation to their behavioral and physiological development. Poult. Sci..

[2] Regmi P., Deland T.S., Steibel J.P., Robison C.I., Haut R.C., Orth M.W., Karcher D.M. (2015). Effect of rearing environment on bone growth of pullets. Poult. Sci..

[3] Tactacan G.B., Guenter W., Lewis N.J., Rodriguez-Lecompte J.C., House J.D. (2009). Performance and welfare of laying hens in conventional and enriched cages. Poult. Sci..

[4] Li Z.Y., Wang C.Y., Li B.M., Shi Z.X., Zheng W.C., Teng G.H. (2020). Concentration and size distribution of particulate matter in a new aviary system for laying hens in China. J. Air Waste Manag. Assoc..

[5] Campbell D.L.M., Ali A.B.A., Karcher D.M., Siegford J.M. (2017). Laying hens in aviaries with different litter substrates: Behavior across the flock cycle and feather lipid content. Poult. Sci..

[6] Banhazi T.M., Seedorf J., Laffrique M., Rutley D.L. (2008). Identification of the risk factors for high airborne particle concentrations in broiler buildings using statistical modelling. Biosyst. Eng..

[7] Le Bouquin S., Huneau-Salauen A., Huonnic D., Balaine L., Martin S., Michel V. (2013). Aerial dust concentration in cage-housed, floor-housed, and aviary facilities for laying hens. Poult. Sci..

[8] Mazaheri A., Lierz M., Hafez H.M. (2005). Investigations on the pathogenicity of *Erysipelothrix rhusiopathiae* in laying hens. Avian Dis..

[9] Shepherd T.A., Zhao Y., Li H., Stinn J.P., Hayes M.D., Xin H. (2015). Environmental assessment of three egg production systems—Part II-ammonia, greenhouse gas, and particulate matter emissions. Poult. Sci..

[10] Zhao Y., Shepherd T.A., Li H., Xin H. (2015). Environmental assessment of three egg production systems—Part I: Monitoring system and indoor air quality. Poult. Sci..

[11] Martin C.D., Mullens B.A. (2012). Housing and dustbathing effects on northern fowl mites (*Ornithonyssus sylviarum*) and chicken body lice (*Menacanthus stramineus*) on hens. Med. Vet. Entomol..

[12] Vanliere D. (1992). Dustbathing as related to proximal and distal feather lipids in laying hens. Behav. Process..

[13] Olsson I.A.S., Keeling L.J. (2005). Why in earth? Dustbathing behaviour in jungle and domestic fowl reviewed from a tinbergian and animal welfare perspective. Appl. Anim. Behav. Sci..

[14] Alvino G.M., Tucker C.B., Archer G.S., Mench J.A. (2013). Astroturf as a dustbathing substrate for laying hens. Appl. Anim. Behav. Sci..

[15] Pokharel B.B., Boecker I., Kwon I.Y., Jeyachanthiran L., McBride P., Harlander-Matauschek A. (2018). How does the presence of excreta affect the behavior of laying hens on scratch pads?. Poult. Sci..

[16] Guinebretière M., Michel V., Arnould C. (2015). Dustbathing, pecking and scratching behaviours of laying hens in furnished cages are enhanced by the presence of rubber mats and litter distribution. Appl. Anim. Behav. Sci..

[17] Lee H.-W., Louton H., Schwarzer A., Rauch E., Probst A., Shao S., Schmidt P., Erhard M.H., Bergmann S. (2016). Effects of multiple daily litter applications on the dust bathing behaviour of laying hens kept in an enriched cage system. Appl. Anim. Behav. Sci..

[18] Scholz B., Kjaer J.B., Urselmans S., Schrader L. (2011). Litter lipid content affects dustbathing behavior in laying hens. Poult. Sci..

[19] Sandilands V., Baker L., Donbavand J., Brocklehurst S. (2021). Effects of different scratch mat designs on hen behaviour and eggs laid in enriched cages. Animals.

[20] Zhang Q. (2023). Study on Sand Bath Behavior of Laying Hens and Its Facilities in Large Cage Aviary Unit System. Master’s Thesis.

[21] Nørgaard-Nielsen G. (1997). Dustbathing and feather pecking in domestic chickens reared with and without access to sand. Appl. Anim. Behav. Sci..

[22] Moesta A., Knierim U., Briese A., Hartung J. (2008). The effect of litter condition and depth on the suitability of wood shavings for dustbathing behaviour. Appl. Anim. Behav. Sci..

[23] Knierim U., Schrader L., Steiger A. (2006). Alternative Legehennenhaltung in Der Praxis: Erfahrungen, Probleme, Lösungsansätze.

[24] Louton H., Bergmann S., Reese S., Erhard M.H., Rauch E. (2016). Dust-bathing behavior of laying hens in enriched colony housing systems and an aviary system. Poult. Sci..

[25] van Liere D.W. (1992). The significance of fowls’ bathing in dust. Anim. Welf..

[26] Roll V.F.B., Levrino G.A.M., Briz R.C. (2008). Rearing system and behavioural adaptation of laying hens to furnished cages. Cienc. Rural.

[27] Wall H., Tauson R., Elwinger K. (2008). Effects of litter substrate and genotype on layers’ use of litter, exterior appearance, and heterophil:lymphocyte ratios in furnished cages. Poult. Sci..

[28] Sanotra G.S., Vestergaard K.S., Agger J.F., Lawson L.G. (1995). The relative preferences for feathers, straw, wood-shavings and sand for dustbathing, pecking and scratching in domestic chicks. Appl. Anim. Behav. Sci..

[29] Grebey T.C., Ali A.B.A., Swanson J.C., Widowski T.M., Siegford J.M. (2020). Dust bathing in laying hens: Strain, proximity to, and number of conspecifics matter. Poult. Sci..

[30] Li M.X. (2017). The Effect of Cement Mortar Ground Covered with Thin Sand on the Physical Condition and Behavior of Laying Hens. Master’s Thesis.

[31] Vestergaard K. (1982). Dust-bathing in the domestic fowl—Diurnal rhythm and dust deprivation. Appl. Anim. Ethol..

[32] van Liere D.W. (1991). Function and Organization of Dustbathing in Laying Hens.

[33] Campbell D.L.M., Makagon M.M., Swanson J.C., Siegford J.M. (2016). Litter use by laying hens in a commercial aviary: Dust bathing and piling. Poult. Sci..

[34] Olsson I.A.S., Duncan I.J.H., Keeling L.J., Widowski T.M. (2002). How important is social facilitation for dustbathing in laying hens?. Appl. Anim. Behav. Sci..

[35] Carol Petherick J., Seawright E., Waddington D., Duncan I.J.H., Murphy L.B. (1995). The role of perception in the causation of dustbathing behaviour in domestic fowl. Anim. Behav..

[36] Yin P., Tong Q., Li B.M., Zheng W.C., Wang Y., Peng H.Q., Xue X.L., Wei S.Q. (2024). Spatial distribution, movement, body damage, and feather condition of laying hens in a multi-tier system. Poult. Sci..

[37] Petherick J.C., Duncan I.J.H. (1989). Behaviour of young domestic fowl directed towards different substrates. Br. Poult. Sci..

